# Lipoprotein receptors: A little grease for enveloped viruses to open the lock?

**DOI:** 10.1016/j.jbc.2024.107849

**Published:** 2024-09-30

**Authors:** François-Loïc Cosset, Solène Denolly

**Affiliations:** 1 CIRI – Centre International de Recherche en Infectiologie, Université de Lyon, Université Claude Bernard Lyon 1, Inserm, U1111, CNRS, UMR5308 ENS de Lyon, Lyon, France; 2 Centre de Recherche en Cancérologie de Lyon, Inserm U1052-CNRS UMR5286, Université de Lyon, Université Claude Bernard Lyon1, Centre Léon Bérard, Lyon, France

**Keywords:** virus receptor, viral entry, enveloped virus, lipoprotein receptor, lipoprotein, LDL-R, apolipoprotein, ApoE, lipid transfer, antiviral

## Abstract

Several studies recently highlighted the role of lipoprotein receptors in viral entry. These receptors are evolutionarily ancient proteins, key for the transport of lipids as well as other signaling molecules across the plasma membrane. Here, we discuss the different families of lipoprotein receptors and how they are hijacked by enveloped viruses to promote their entry into infected cells. While the usage of lipoprotein receptors was known for members of the *Flaviviridae* family and vesicular stomatitis virus, the last 4 years have seen the discovery that these receptors are used by many genetically unrelated viruses. We also emphasize how viral particles interact with these receptors and the possible targeting of these host factors as antiviral strategies.

Viruses are obligate intracellular pathogens that rely on the host cell machinery for their replication and transmission. Enveloped viruses are characterized by a lipid bilayer incorporating one or more viral surface glycoproteins. To infect a new cell, the enveloped viral particles need to attach to the cell surface, *via* non-covalent interactions between host factors at the plasma membrane and exposed components of the viral particles (glycans of proteins associated to viral particles, specific phospholipids, cellular factors associated to viral particles, or virus-encoded surface glycoproteins). After binding to these host factors, often designated as “virus receptors”, viral particles can penetrate cells *via* two different routes: either an endocytic one or a non-endocytic one. Irrespective of the route, the cell entry process subsequently involves a fusion step between viral and cell membranes, which is mediated by conformational changes of viral surface glycoproteins and is followed by an “uncoating” step whereby the viral ribonucleoprotein or nucleocapsid is released from its membrane envelope. For the non-endocytic route, viral entry relies on the fusion of the membrane of viral particles with the plasma membrane. For the endocytic route, viral entry relies on internalization, which is mediated by different cellular processes (such as *e.g.,* transport in clathrin-coated vesicles or micropinocytosis), before the fusion of viral membranes with the membrane of endosomes. These steps are often referred to as “post-binding events” to mention both viral endocytosis, membrane fusion, and uncoating.

Viruses have evolved to use various types of cell surface receptors to initiate their entry into cells. Among them, several lipoprotein receptors were recently identified as key factors for the entry of a wide array of genetically unrelated viruses. Lipoprotein receptors are proteins expressed at the cell surface and are key players in lipid transport. More particularly, they are key for the transfer of lipids as well as of other essential extracellular signaling molecules across the plasma membrane. Here, we review how different families of viruses hijack the lipoprotein receptors during their entry steps.

## Lipoprotein receptors

Plasma lipoproteins are key transporters of neutral lipids, which are essential to feed various tissues for energy and metabolism. They are composed of a hydrophobic core containing neutral lipids, *i.e.* triglycerides and cholesterol esters, surrounded by a monolayer of surface lipids, *i.e.*, amphipathic phospholipids, free cholesterol, and they incorporate proteins called apolipoproteins (apo), such as the non-exchangeable apoB apolipoprotein and several exchangeable apolipoproteins, like apoE (1). Circulating lipoproteins are categorized according to their lipid and protein contents and thus their density within three main categories: the very low-density lipoproteins (VLDL), which are synthesized in the liver; the low-density lipoproteins (LDL), which are the results of lipid transfer and hydrolysis of VLDL in the bloodstream, and the high-density lipoproteins (HDL), which originates from the maturation of pre-β HDL in the bloodstream.

Lipid transfer from lipoproteins across plasma membrane requires the action of several types of lipoprotein receptors which can be classified into two groups: the endocytic receptors and the lipid uptake receptors. For the first category, the receptors bind to the lipoproteins, which mediate their internalization and subsequent lysosomal delivery; this category includes the members of the low-density lipoprotein receptor (LDL-R) family. For the second category, the receptors bind to the lipoproteins but the lipid molecules are directly transferred to the plasma membrane without cellular uptake of other components of the lipoproteins; this category includes the scavenger type B receptors.

## The LDL-R family

**Figure 1:**
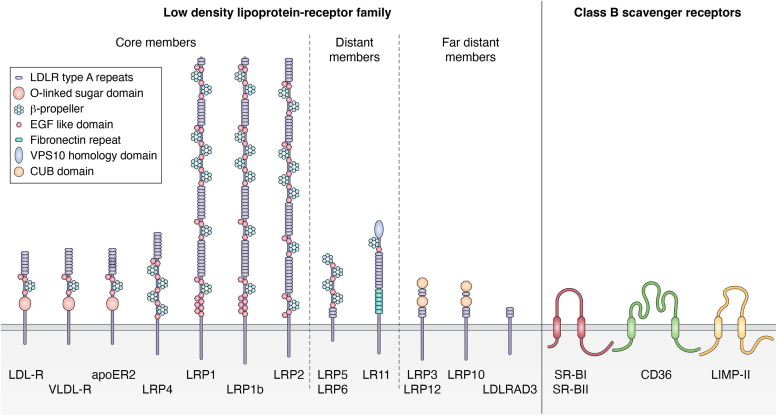
**Lipoprotein receptors**.

The LDL-R family consists of a class of cell surface endocytic receptors that are structurally and functionally linked to the patriarch of the family, the LDL-R (Fig. 1). The LDL-R was the first cellular receptor for lipoproteins to be identified (2) and this discovery was awarded the Nobel Prize in Physiology or Medicine in 1985 (Brown and Goldstein). The LDL-R family includes seven core members (Table 1) with, in addition to the LDL-R itself, the LDL receptor-related protein (LRP1) (3), LRP2 or Megalin (4), the VLDL receptor (5), the ApoE receptor-2 (ApoER2, aka LRP8) (6, 7), LRP1b (8), and LRP4 or MEGF7 (9) but also more distinct members including LRP5 (10, 11), LRP6 (12) or LRP11 (13, 14). Structurally, all the core members are composed of a combination of two domains: the ligand binding domains, called LDL-R type A repeats (or LA repeats), and the epidermal growth factor (EGF)-precursor homology domains, which are responsible for the pH-dependent release of ligands in endosomes and composed of EGF-like repeats (or EGF repeats) and β-propeller repeats. The LDL-R, VLDL-R, and LRP8 also contain O-linked sugar domains. The cytosolic tails of these molecules are less well conserved than their ectodomains but all members contain at least one NPxY “tyrosine” motif that is key for signal transduction (15) and endocytosis (16). Some very distant members of the LDL-R family have also been identified and have only LDL-R type A repeats, like for the LDLRAD3 (17).

**Table 1:** The core members of the LDL-R family

Receptors	Expression	Ligands
LDL-R	Ubiquitous	apoB, apoE
LRP1	Ubiquitous, abundant in liver and brain	>100: apoE, proteases, protease inhibitor complexes, extracellular matrix proteins, growth factors, heat shock proteins, …
LRP2 (Megalin)	Mainly in kidney	>30: apoE, apoB, hormones, enzymes and inhibitors, …
VLDL-R	Adipose tissues, skeletal muscle, heart, endothelial cells of capillaries and small arterioles	apoE, Reelin
ApoER2 (LRP8)	Brain, testis, placenta	apoE, Reelin
LRP1b	Brain, thyroid gland, skeletal muscle	Fibrinogen, vitronectin, apoE
LRP4 (MEGF7)	Brain	Agrin

The members of the LDL-R family recognize a wide variety of structurally and biochemically different ligands that, for some receptors, can be shared (Table 1). Some ligands, such as *e.g.*, Agrin or Reelin that bind LRP4, VLDL-R, and apoER2 typically belong to the extracellular matrix of different tissues, whereas other ligands belong to the family of apolipoproteins, such as apoE and apoB that are well-characterized ligands for LDL-R, VLDL-R, and apoER2. Importantly, there is a wide variation in the number and types of ligands used by the LDL-R family members, with LRP1 being able to bind over 100 different molecules (Table 1). This highlights the fact that members of the LDL-R family perform other or additional functions than lipid transfer; in particular, some members play a role in signal transduction (18).

**Figure 2:**
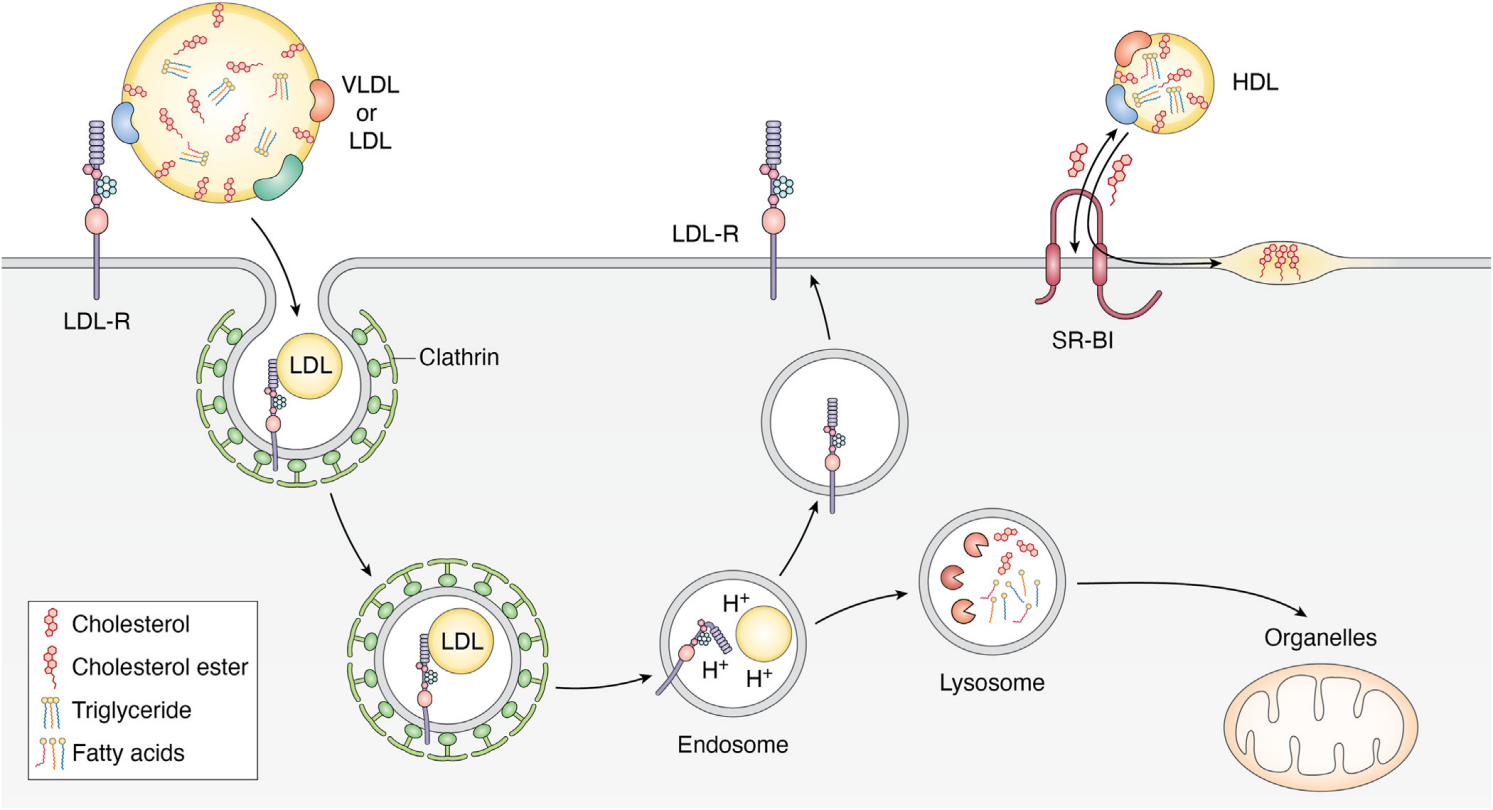
**Mode of action of lipoprotein receptors.** (*Left*) Upon binding of LDL or VLDL *via* apoE and/or apoB, the lipoprotein-receptor complex undergoes clathrin-mediated endocytosis. Upon acidification of the endosomes, conformational change of LDL-R occurs with an interaction of LA4-5 with beta propeller, allowing the release of the lipoprotein. The lipoprotein is directed to lysosome, allowing the release of free cholesterol and fatty acids, while LDL-R is recycled back to the plasma membrane. (*Right*). On the other hand, upon binding of HDL to SR-BI, the transfer of cholesterol ester to plasma membrane or the bi-directional transfer of cholesterol are mediated *via* a tunnel in SR-BI.

Perhaps the best-characterized function of ligand/receptor interaction within the LDL-R family members leading to LDL endocytosis is the well-known example of the LDL-R itself. Indeed, recognition of apolipoproteins such as apoE and apoB carried by lipoproteins with the receptor at the cell surface leads to their internalization *via* clathrin-coated pits (Fig. 2). Lipoproteins are then released from their receptors at acidic pH, around pH6, which corresponds to the luminal pH of sorting endosomes, *via* a conformational change of LDL-R ectodomain (19). The LDL-R is recycled back to the plasma membrane, while lipoproteins travel to lysosomes where they are degraded to release amino acids, cholesterol, and fatty acids. The same type of mechanism could be extended to the other members of the family and their ligands. In addition, these receptors can act as signal transducers through interaction with cytosolic adapters (20).

## The scavenger type B receptors

Class B scavenger receptors include several receptors, such as SR-BI (21), SR-BII (22), an isoform of SR-BI, lysosomal integral membrane protein II (LIMP-II; a lysosomal protein) (23) and CD36 (24). These molecules are characterized by two transmembrane domains flanking an extracellular/luminal loop, whose amino- and carboxy-termini are located in the cytosol. SR-BI is expressed in numerous tissues, including the liver, and can bind a large variety of ligands including HDL, LDL, and VLDL as well as several proteins, underscoring its multiple functions. However, the main physiological function of SR-BI is the binding to HDL and, *via* lipids and apolipoproteins, the uptake of cholesterol ester (21) that is directly incorporated within the plasma membrane bilayer (Fig. 2). In addition, SR-BI mediates the bidirectional flux of cholesterol between HDL and cells (25). CD36 also binds different classes of ligands including oxidized phospholipid moieties found in oxidized low-density lipoproteins (oxLDL) (26) as well as native lipoproteins (27) and is involved in the uptake of long-chain free fatty acid (28).

Based on structural studies on LIMP-II, the 3D modeling of SR-BI showed that a large lipophilic tunnel traverses the entire SR-BI ectodomain through which cholesterol ester is transferred from the bound lipoproteins inside the leaflets of the plasma membrane (29, 30). This direct transport is believed to locally enrich the plasma membrane in lipids and, therefore, its composition and biochemical properties. A similar type of tunnel was modeled for CD36 and proposed to facilitate the transfer of fatty acids, though, in contrast, the uptake of oxLDL occurs *via* endocytosis of the receptor (31).

## Enveloped viruses that use lipoprotein receptors

### Hepatitis C virus, not a unique case anymore?

Hepatitis C virus (HCV) is a (+) strand RNA virus, that causes liver diseases such as cirrhosis and hepatocellular carcinoma in humans. HCV particles have the specific feature of being associated with neutral lipids and apolipoproteins, such as apoE, apoB, and other apolipoproteins (32), which confers its broad range of buoyant densities (from 1.00 to 1.35). Because of this, the role of lipoprotein receptors in HCV entry has been studied in much detail. The LDL-R was one of the first receptors identified for HCV (33) based on binding assays with patient-derived viral particles. Yet, its role in HCV entry remains controversial since further studies indicated that HCV uptake by LDL-R could lead to unproductive infection (34). Rather, the LDL-R might regulate the intracellular lipid content (34), which is necessary to promote HCV replication. SR-BI was later identified as an attachment factor for E2 (one of the two HCV surface glycoproteins) in CD81-negative cells (35); CD81 being the main and essential receptor for HCV (36). SR-BI plays a critical role in HCV entry by allowing the initial attachment of the viral particles to the cell surface. This attachment occurs *via* interaction with apoE that is present at the surface of viral particles (37, 38, 39), which may mimic some features of the interactions of SR-BI with lipoproteins. Moreover, SR-BI also directly binds to the HCV glycoprotein E2 (35, 38, 40) and this interaction was proposed to prime particles for binding to CD81 by inducing a conformational change of E2 that leads to unmasking the CD81-binding site. Finally, SR-BI also promotes post-binding entry events in an E2-independent manner although this is related to its lipid transfer activity (39) and the concomitant presence of HDLs (41, 42, 43), which may occur by altering the local composition and fluidity of the target membrane with which HCV must fuse.

Interestingly, more recent studies indicated that both LDL-R and VLDL-R may compensate for the lack of SR-BI for HCV entry (44) or that VLDL-R can induce an alternative entry pathway (45), suggesting that lipoprotein receptors might play at least partial redundant roles in HCV entry. Indeed, the receptor choice might depend on the density of the viral particles (39, 44), suggesting that HCV particles mimic the different classes of lipoproteins to use the related receptors.

**Figure 3:**
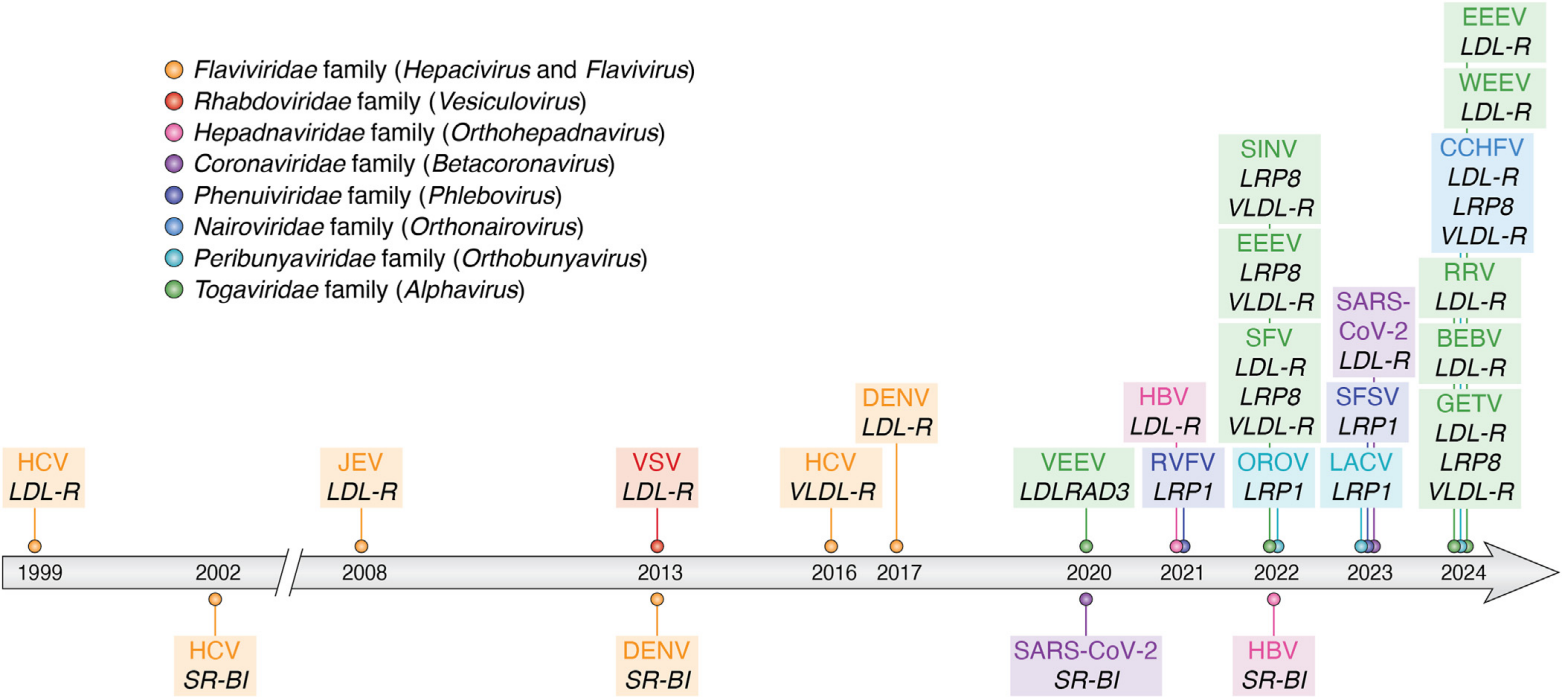
**Timeline of discoveries of lipoprotein receptors as virusreceptors.** In *orange* are depicted members of the *Flaviviridae* family: hepatitis C virus (HCV), an *Hepacivirus*, and Japanese encephalitis virus (JEV) and Dengue virus (DENV), two *Flaviviruses*. In *pink* is depicted vesicular stomatitis virus (VSV), a *Vesiculovirus*. In *red* is depicted hepatitis B virus (HBV), an *Orthohepadnavirus*. The *green* color corresponds to members of *Togaviridae* family from the *Alphavirus genus*: Venezuelan equine encephalitis virus (VEEV), Sindbis virus (SINV), Eastern equine encephalitis virus (EEEV), Semliki forest virus (SFV), Western equine encephalitis virus (WEEV), Ross river virus (RRV), Getah virus (GETV), Bebaru virus (BEBV). In *blue* are depicted members of *Bunyaviricetes class* with members of *Peribunyaviridae* family/*Orthobunyavirus* genus with Oropouche virus (OROV) and La Crosse virus (LACV), members of *Nairoviridae* family/*Orthonairovirus* genus with Crimean-Congo hemorrhagic fever virus (CCHFV) and members of *Phenuiviridae* family/*Phlebovirus* genus with Rift valley fever virus (RVFV), Sandfly fever Sicilian virus (SFSV).

For nearly 15 years, HCV was considered as the sole virus that relies on lipid transfer receptors to mediate its entry into cells, in tight relation to what is considered as its unique and original feature, *i.e.*, the capacity of its viral particles to incorporate and/or associate with neutral lipids and apolipoproteins as a so-called lipo-viro-particle (LVP). Yet, it later appeared that while they may not form LVPs, alternative viruses from a broad range of distinct families and orders use lipid transfer receptors for cell entry (Fig. 3, Table 2).

**Table 2:** The usage of lipoprotein receptors in viral entry

Genus	Virus	Other receptor(s)	Lipoprotein receptor	Ligand	References
*Hepacivirus*	HCV	CD81, OCLN, CLDN…	LDL-R	apoE	(33, 34)
			VLDL-R		(44, 45)
			SR-BI	apoE, E2	
*Flavivirus*	DENV	DC-SIGN, TIM, AXL,…	LDL-R		(57)
			SR-BI	apoA-I	(68)
	JEV	DC-SIGN, TIM, HSP90, …	LDL-R	E	(58, 59)
*Vesiculovirus*	VSV		LDL-R	G	(46, 47, 49)
			Other, LRP1?		(46, 49, 52)
*Orthonairovirus*	CCHFV	Nucleolin, DC-SIGN	LDL-R	Gc, apoE	(62, 63, 64)
			VLDL-R		(64)
			LRP8	apoE?	(63)
*Phlebovirus*	RVFV	DC-SIGN	LRP1	Gn	(51, 52, 103)
	SFSV		LRP1		(52)
*Orthobunyavirus*	OROV		LRP1		(50)
	LACV		LRP1	Gn	(52)
*Hepadnavirus*	HBV	NTCP	LDL-R		(55)
			SR-BI	preS1	(69)
*Alphavirus*	GETV	MXRA8	LDL-R	E1/E2	(60)
			LRP8		(60)
			VLDL-R		(60)
	SFV	MXRA8	LDL-R	E1/E2	(54, 60, 61)
			LRP8	E1/E2	(54)
			VLDL-R	E1/E2	(54, 81)
	BEBV	MXRA8	LDL-R	E1/E2	(60)
	RRV	MXRA8	LDL-R	E1/E2	(60)
	EEEV		LRP8	E1/E2	(54)
			VLDL-R	E1/E2	(54, 78)
			LDL-R		(61)
	WEEV	MXRA8	LDL-R		(61)
	SINV	MXRA8	LRP8	E1/E2	(54)
			VLDL-R	E1/E2	(54)
	VEEV		LDLRAD3	E1/E2	(53, 79, 80)
*Betacoronavirus*	SARS-CoV-2	ACE2	LDL-R	apoE	(56)
			SR-BI	S-HDL	(67)

BEBV, Bebaru virus; CCHFV, Crimean-Congo hemorrhagic fever virus; DENV, Dengue Virus; EEEV, Eastern equine encephalitis virus; GETV, Getah virus; HBV, Hepatitis B virus; HCV, Hepatitis C virus; JEV, Japanese encephalitis virus; LACV, La Crosse virus; OROV, Oropouche virus; RRV, Ross river virus; RVFV, Rift Valley fever Virus; SARS-CoV-2, Severe acute respiratory syndrome coronavirus; SFSV, Sandfly fever Sicilian virus; SFV, Semliki forest virus; SINV, Sindbis virus; VEEV, Venezuelan equine encephalitis virus; VSV, Vesicular stomatitis virus; WEEV, Western equine encephalitis virus.

### LDL-R family: pan-virus receptors?

After a particularly long search, the LDL-R was identified as the main receptor for the vesicular stomatitis virus (VSV), in line with the broad tropism of this virus (46, 47). This discovery came from the original observation that interferon inhibits VSV infection by inducing the secretion of a soluble form of LDL-R (48). The formal identification of LDL-R was then achieved by using LDL-R deficient cells and blocking antibodies (46). However, some other members of the LDL-R family can also serve as alternative receptors for VSV, though with a lower efficiency than for LDL-R. Indeed, VSV infection of LDL-R negative cells can be inhibited by the addition of exogenous Receptor-associated protein (RAP), which is a common chaperone of LDL-R family members that prevents the binding of ligands to these receptors (46, 49).

It was only after several years and more particularly during the last 4 years that it was discovered that LDL-R family members act as entry factors if not receptors for a plethora of genetically unrelated viruses (Fig. 3, Table 2). By screening unbiased genome-wide libraries of CRISPR/Cas9-based single-guide RNAs or of knockout haploid mouse embryonic stem cells, LRP1 was first shown to act as a receptor for two members of the *Peribunyaviridae* and *Phenuiviridae* families of the *Bunyaviricetes* class: the Oropouche virus (OROV) (50) and the Rift Valley fever virus (RVFV) (51, 52). Later on, it appeared that other members of the LDL-R family are used by viral pathogens from distinct orders to promote cell entry, like the LDLRAD3 for the Venezuelan equine encephalitis virus (VEEV), an alphavirus (53), the ApoER2 for several other alphaviruses such as Semliki Forest virus (SFV), Eastern equine encephalitis virus (EEEV), and Sindbis virus (SINV) (54), and the VLDL-R for the latter alphaviruses (54) or, as above discussed, HCV (44, 45). Likewise, the usage of LDL-R itself was shown to be shared by several other viruses than HCV or VSV, genetically unrelated, including hepatitis B virus (HBV) (55), SARS-CoV-2 (56), Dengue virus (DENV) and Japanese encephalitis virus (JEV), two flaviviruses (57, 58, 59), several alphaviruses (60, 61) and the Crimean Congo hemorrhagic fever virus (CCHFV), an orthonairovirus from the *Bunyaviricetes* class (62, 63, 64). The latter and simultaneous discovery of the CCHFV receptors included not only the LDL-R but also other members of the family, such as the VLDL-R (64) and LRP8 (63), which followed a long period of suspense after the early proposition that the human C-type lectin DC-SIGN and the nuclear factor Nucleolin were proposed to be involved in CCHFV entry (65, 66), although it was clear that they were not sufficient for CCHFV entry.

In comparison to the LDL-R family, there are far fewer viruses that use the scavenger type B receptors (Table 2). This could be due to their different mechanism of lipid transfer as, in contrast to LDL-R family members, this does not require their internalization (Fig. 2), which indicates that they may not be used, at least alone, by viruses that necessitate endocytosis to promote cell entry. Viruses using SR-BI include HCV, as above discussed (35, 40), SARS-CoV-2 (67), DENV (68) and HBV (69). Several reports have highlighted that some of these viruses may use SR-BI to achieve post-binding functions that are required for cell entry, at the membrane fusion step or other steps (39, 41, 42, 43). Moreover, it is remarkable that two viral pathogens that are so different, *i.e.*, HCV and SARS-CoV-2, seem to exploit SR-BI properties to mediate similar entry functions. This includes not only the attachment of their viral particles but also the lipid-transfer functions of SR-BI, since blocking of SR-BI with antibodies and of lipid transfer with pharmacological SR-BI antagonists could readily prevent virus entry as well as the HDL-mediated enhancement of infection for either virus type (42, 43, 67, 70).

## Interaction between viruses and lipoprotein receptors

### Interaction of LDL-R family members with viral glycoproteins: a conserved mechanism?

The identification of members of the LDL-R family as virus receptors led to the study of the ligands used by the viruses and the interaction mechanisms.

**Figure 4:**
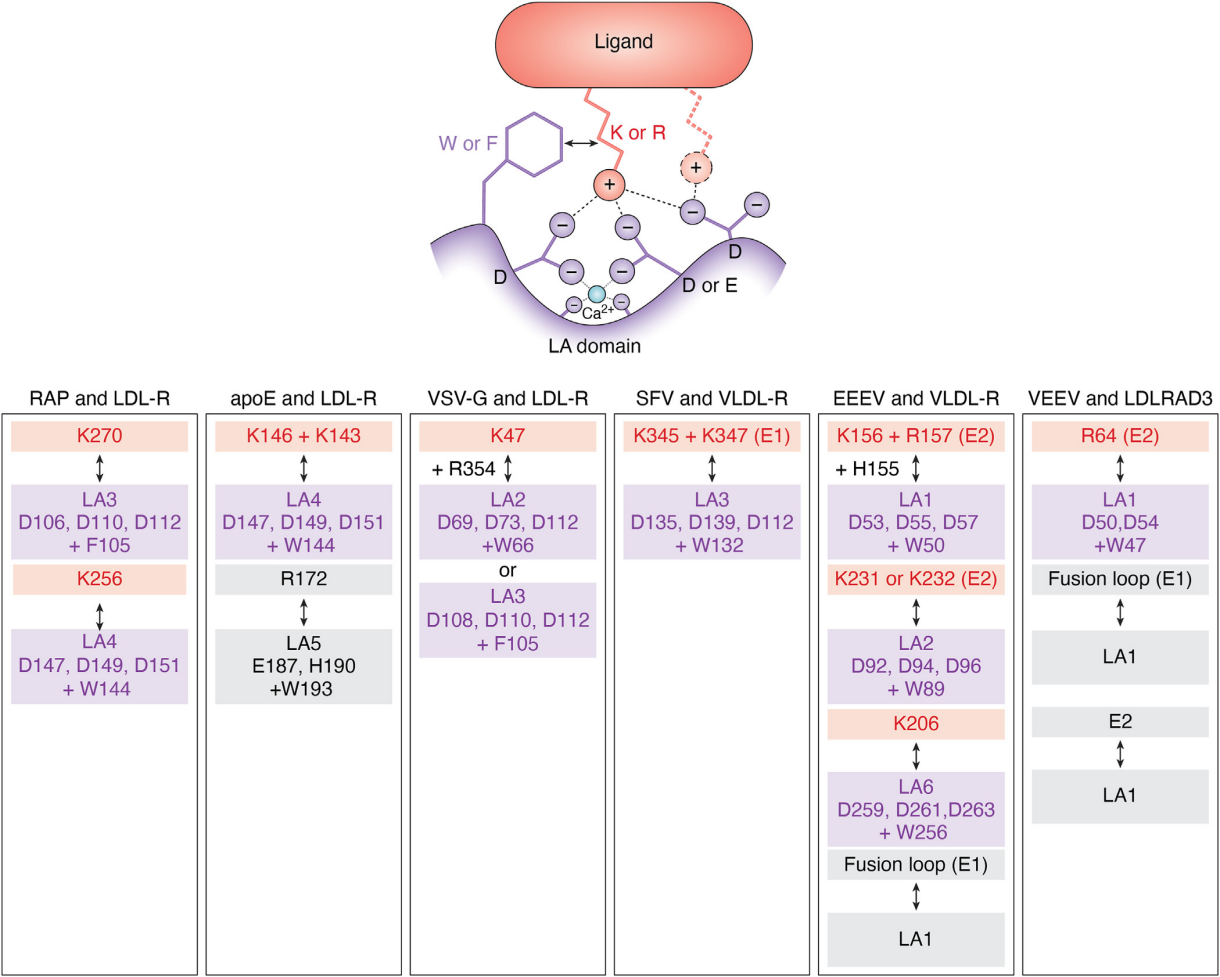
**Structural insights of interaction between LDL-R family and ligands.** The mechanism of interaction between the viral glycoproteins and member of the LDL-R family is relatively well conserved with the usage of the Ca^2+^ coordinating cage and a close aromatic residue on the receptor side (*violet*) and the lysine residue on the lipoprotein/viral side (*red*). Other interface regions can also be involved (*grey*).

First of all, the structure of the third domain of RAP in complex with two modular regions of LDL-R (LA repeats 3 and 4) provided indications on the mode of recognition of a physiologic LDL-R ligand by the LA modules for different proteins of the LDLR-family (71) and suggested that the mode of interaction could be conserved for the other ligand/receptor complexes (Fig. 4). The contact site between each LA and the RAP domain is formed by electrostatic interactions between conserved acidic residues of LA, within a calcium coordination sphere, and a basic lysine residue in RAP helices. The binding is reinforced by a hydrophobic interaction between an aromatic residue of either LA domain with the aliphatic region of the lysine.

Likewise, for apoE, a cluster of basic amino acids between residues 140 and 160, particularly two lysines at position 143 and 146 (72), together with another arginine at position 172 (R172) (73), were identified as LDL-R binding site (Fig. 4). LDL-R uses LA4-5 for this interaction (74, 75).Therefore, a docking model proposed a mode of LDL-R interaction for apoE similar to that of RAP with two key lysine residues (71), which was later confirmed by the interaction of these two lysines with LA4 (76). It was further proposed that R172 interacts with LA5 through residues close to the Ca^2+^ coordination sites (76). Interestingly, apoE is only able to bind LDL-R in its lipid-bound forms, in contrast to Lrp1 and VLDL-R (77), which is probably because i) only the conformation of the lipid-bound apoE form allows the positioning of R172 to generate the additional contact with the LA domain, thus inducing a complete binding site by improving the charged environment, ii) the presence of several molecules of apoE on lipoproteins creates multivalent ligands on the same lipoprotein, which thus increases the avidity of apoE to LDL-R.

In line with this model of native receptor/ligand interaction, LDL-R was shown to directly bind the VSV glycoprotein (VSV-G) *via* the LA repeats two and 3 of the LDL-R, which, like apoE, also involves two basic residues on VSV-G (49), a lysine (K47) interacting directly with the Ca^2+^ coordinating residues and an arginine (R354) interacting with the main chain of the LA domains (Fig. 4).

Several other studies provided structural information regarding the binding of LDL-R family members to the glycoproteins E1 and E2 of alphaviruses (Fig. 4). For EEEV and VLDL-R as well as for VEEV and LDLRAD3, the interaction between either glycoprotein and the receptors engage several sites on E1/E2, including a cleft structural motif formed by two adjacent E2–E1 heterodimers in the trimeric spike and several LA domains of the receptors (78, 79, 80). Especially, EEEV interacts with VLDL-R by engaging LA1, LA2, LA3, LA5 and LA6 domains (all having in common a tryptophan in the Ca^2+^ coordination complexes) (78). More specifically, two sites of interactions involve Ca^2+^ coordinating residues of LA1 and LA2 and basic residues of E2 of EEEV, though another site involves a different mechanism with the interaction of fusion loop in E1 and different residues of LA1. An additional interacting site could be observed for some EEEV strains, since LA3, LA5, and LA6 can also make a contact site with E2, again using Ca^2+^ coordination sites, and other basic residues of E2 (78). For VEEV and LDLRAD3-D1, D1 corresponds to the most distal LA domain, one interaction involves the fusion loop of E1 with D1, two other interaction sites involve E2 basic residues (arginine) and the Ca^2+^ coordination sphere in D1 for the first one and other residues in E2 and D1 on for the second one (79, 80). Finally, based on the structural similarity between D1 and LA4-LA5 domains of LDL-R, residues in the Ca^2+^ coordinating area were also predicted for interaction between LDL-R and Getah virus (GETV) (60).

Interestingly, the analysis of the structures generated indicates that the mechanism is different for SFV, though SFV can also bind to multiple LA domains of VLDL-R, with the highest affinity for LA3. Moreover, Cryo-EM structures indicate that VLDL-R LA3 only binds SFV E1 (81). Yet, again, this interaction involves the classical interaction of LDL-R ligand *via* three Ca^2+^ coordinating acidic residues with basic residues of E1 (81).

For all the above-mentioned viruses, it is also clear that, despite this conserved mechanism of interaction with lipoprotein receptors, there are additional residues that are involved in the stabilization of the interaction between the ligands and the receptors.

Regarding bunyaviruses, there is currently a lack of structural evidence to provide a complete picture of the interaction between receptors and viral particles. The Gn proteins of RVFV or OROV were shown to be involved in binding to LRP1 (50, 51), while the Gc glycoprotein was identified as involved in the interaction of CCHFV particles with LDL-R (62, 63).

### Apolipoproteins: partners in crime of viral particles to promote entry?

While the above considerations indicated that the surface glycoproteins of the enveloped viruses that use members of the LDL-R family are *bona fide* ligands of these receptors, it became clear that, at least for a subset of these viruses, intrinsic properties of the cell type used as virus producer cells could modulate their usage for entry. Specifically, the blocking of LDL-R in Huh-7.5 target cells with an LDL-R antibody had less impact on the infectivity of CCHFV particles produced in HEK-293T cells than for those produced in Huh-7.5 cells (64), which led to a question about the presence of a cellular imprinting factor on the virions produced. Of outstanding interest, CCHFV particles were found to incorporate apoE, as evidenced by co-immuno-precipitation of viral RNA and Gn/Gc glycoproteins by apoE antibodies as well as by electron microscopy experiments and immunogold labeling with anti-Gn or anti-apoE antibodies (64). Subsequently, a direct link between recruitment of apoE on CCHFV particles, though not on HAZV—*i.e.*, Hazara virus, another member of the genus—and LDL-R usage was revealed, since apoE, which serves as a ligand for several lipoprotein receptors (Table 1), could initiate or stabilize the interaction of CCHFV particles with the LDL-R (64) or with apoER2 (63), as shown by the loss of receptor dependency upon apoE blocking or knock-down assays.

However, this striking feature is not unprecedented as it resembles that observed some time ago for HCV, for which incorporation of apoE within viral particles was shown to mediate the binding to LDL-R (82) and SR-BI (39). Along the same line, SARS-CoV-2 also incorporates apoE within its viral particles to promote the usage of LDL-R for entry (56). Finally, HBV also incorporates apoE (83, 84) on its particles and is dependent on LDL-R for entry (55), as above discussed, although it remains to be clarified if apoE incorporation on HBV particles implements the usage of LDL-R (Table 2).

ApoE is mainly produced in the liver, brain, adrenal glands, testes, kidneys, and macrophages. As an exchangeable plasma apolipoprotein, apoE can be transferred between lipoproteins such as chylomicrons, VLDLs, intermediate density lipoproteins (IDL), and a subgroup of HDLs, depending on their lipid composition and the resulting affinity of apoE to these lipid carriers, although apoE can also be found in lipid-free, soluble form in the bloodstream. Physiologically, like other exchangeable apolipoproteins that are detected on some enveloped viruses (*e.g.*, apoA-I, apoC-I, apoC-II, apoC-III), apoE has three major functions in the metabolism of lipoproteins: i) it stabilizes their structure and contributes to their solubilization, ii) it interacts with their receptors and contributes to their clearance, and iii) it acts as cofactor for specific enzymes involved in lipoprotein metabolism. In addition, apoE plays a key role in lipoprotein morphogenesis in hepatocytes but also has functions unrelated to lipoproteins associated with exosomes and intracellular endosomal vesicles.

It is therefore not truly surprising that enveloped viruses have usurped exchangeable apolipoproteins to promote specific proviral functions that are not limited to provide ligands (Table 2) for lipid transfer receptors or some heparan sulfate proteoglycans (HSPGs), as discussed above for incorporation of apoE on HCV (32, 82, 85, 86), CCHFV (63, 64) and SARS-CoV-2 (56) particles as well as of apoC-I for HCV (87) or apoA-I on DENV (68) particles. Indeed, other proviral functions of exchangeable apolipoproteins encompass: i) viral morphogenesis and maturation (88, 89, 90) ii) viral pathogenesis (reviewed in (91)), iii) viral evasion from neutralizing antibodies (92, 93)) and iv) post-binding function by enhancement of membrane fusion (reviewed in (93)), the two latter functions being associated to the interaction of a virion-incorporated apolipoprotein with its receptor, as shown for example, with apoC-I and SR-BI for HCV.

## Targeting the lipoprotein receptors: towards an antiviral strategy?

The fact that multiple unrelated viruses use lipoprotein receptors as cell entry receptors or co-factors raises the question of targeting these receptors as a new broad-spectrum antiviral approach. Here again, some early achievements in the field of HCV can be recalled, as they could serve as a basis for this. Indeed, as above discussed, SR-BI has multiple functions during HCV entry, by allowing not only cell binding of viral particles but also crucial post-binding events. The latter appear to be associated with the lipid transfer properties of SR-BI since blocking antibodies as well as small molecule compounds that prevent lipid transfer were shown to inhibit HCV infection (40, 41, 42, 43, 94). On this ground, an SR-BI inhibitor (ITX5061) (70) was tested in a phase 1b clinical trial, ultimately aiming to prevent HCV allograft infection, and showed alteration of plasma HCV RNA kinetics and a substantial reduction in plasma HCV RNA during treatment (95). Overall, even if further investigations of the efficacy of ITX5061 seem to have been discontinued, the results of this study provided a proof of principle that targeting such important receptors should be explored further, particularly owing to the possibility that it could be useful against a wide array of viral pathogens.

### Inhibition of LDL-R family members

As the LDL-R family members do not have catalytic activity, it could be difficult to design inhibitors targeting their function, though there are a few molecules that can inhibit or alter more or less directly their expression and/or cell surface levels. In the case of LDL-R, most studies focused on upregulation of this receptor in order to treat hypercholesterolemia by reducing circulating LDLs, which is an opposite strategy compared to what would be needed for an antiviral treatment. Accordingly, there are only a few studies about the assessment and/or development of molecules that can directly inhibit LDL-R functions. However, Berbamine, a bisbenzylisoquinoline alkaloid isolated from the plant *Berberis amurensis* (used in traditional Chinese medicine) and a known calcium channel or signaling inhibitor that impairs the level of LDL-R at the cell surface (58), was shown to alter viral entry and infection by CCHFV and JEV (58, 64). While this molecule is already used to treat leukopenia in China and Japan, its usage might be difficult, considering that its effect is not specific to the modulation of LDL-R, which might induce a number of side effects. Likewise, pre-treatment of cells with the proprotein convertase subtilisin-like kexin type nine (PCSK9), which, by binding to EGF-like repeats, can inhibit LDL-R recycling and thus LDL-R at the cell surface (96), was shown to inhibit HCV (97) and CCHFV (64) infection. Although both molecules were preclinically evaluated and, for Berbamine, shown to protect mice from a lethal challenge of JEV (58), their usage in patients remains to be assessed.

Another strategy to compete for virus binding would be to use modified lipoproteins, apolipoproteins or other factors that can bind to the receptors and prevent the cellular attachment of viral particles. In line with this possibility, RAP was shown to inhibit viral infection of VSV (46, 49), as well as bunyaviruses such as RVFV (51) and OROV (50). Interestingly, synthetic nano-LDLs were developed for drug delivery (reviewed in (98)) and it would be interesting to see if such approaches could be used for combined antiviral development by blocking the LDL-R in addition to on-site delivery of antivirals.

Finally, it has been known for a long time that monensin, an ionophore that can transport calcium across cell membranes, can decrease LDL-R at cell surface (99) by impairing the conformational changes required for lipoprotein release and thus LDL-R recycling (100). This suggested that targeting Ca^2+^ might also be a possible strategy. While several concerns about the toxicity of monensin in humans were raised, it should be noted that calcium-chelating agents are used in the clinic to treat atherosclerotic cardiovascular disease (101).

### Soluble receptors or LA domains as neutralizing molecules

It is clear that the former therapeutic strategies have serious disadvantages, as they can induce dysfunction of the essential lipid receptors, with a high risk of pathophysiological consequences, even if the duration of treatment of an acute infection is very short as compared with the time needed for the metabolic consequences to appear.

Thus, more specific approaches, such as those that target the interaction between the virus and its receptor without preventing the latter's physiological interaction with lipoproteins, would be necessary but could benefit from an intimate understanding of the molecular properties of the former interaction (Fig. 4).

Several studies highlighted the possibility of using soluble forms of LDL-R and even its LA domains to inhibit viral infection. Soluble receptors were shown to impair HCV (34), VSV (46) and CCHFV (62, 63, 64) infections. Likewise, macromolecules harboring combined LA domains of LDL-R family members could inhibit viral infection *in cellula* for OROV (50), RVFV (51) for VSV (49) and both *in cellula* and *in vivo* for EEEV (61, 78) as well as GETV (60). Interestingly, combinations of several repeats of the selected LA domains were shown to increase the efficiency of the treatment (49, 61). Yet, the efficiency and possible usage of such molecules in humans remain to be evaluated as the environment might be less favorable due to the large amounts of circulating lipoproteins that might decoy such LA domains.

## Concluding remarks

Lipoprotein receptors are hijacked for viral entry by viruses from different families and orders. While this review focused on the role of lipoprotein receptors for cell entry of enveloped viruses, it has to be noted that LDL-R family members can also be coopted for viral replication (34, 52, 102), probably by inducing some transduction signals or metabolic changes, such as upregulation of lipids uptake. Yet, the exponential discovery of lipoprotein receptors as viral entry factors led to new concepts of the emergence of a conserved mechanism among viral families, raising original questions concerning host-pathogen interactions and novel research directions. For example, it is intriguing that, depending on virus species that use lipoprotein receptors, some require incorporation of the natural ligand(s) of these receptors to implement their usage although some others directly bind the receptors *via* their surface glycoproteins. Also, it is interesting to remember that LDL-R family members are expressed in many different cell types and have a high degree of similarity between mammalian species but also arthropod homologs, which could suggest an evolutionary mechanism allowing infection of many host species, especially for arboviruses such as alphaviruses and bunyaviruses. Finally, as there is preliminary evidence that targeting lipoprotein receptors can prevent infection, further research will be needed to determine whether this strategy is feasible for use as a first line of treatment.

## Conflict of interest

The authors declare that they have no conflicts of interest with the contents of this article.
